# Report from the Standards for Pathogen Identification via Next-Generation Sequencing (SPIN) Workshop

**DOI:** 10.1186/s40793-015-0112-z

**Published:** 2015-12-02

**Authors:** Nathan D. Olson, Scott A. Jackson, Nancy J. Lin

**Affiliations:** Biosystems and Biomaterials Division, National Institute for Standards and Technology, 100 Bureau Drive, Gaithersburg, MD 20899 USA

**Keywords:** Genome sequencing, Metrology, Next-generation sequencing, Pathogen identification, Reference materials, Standards

## Abstract

Next-generation sequencing (NGS) is not routinely used in applied settings due to lack of confidence in results. This workshop convened experts to identify measurement challenges impeding NGS implementation and potential standards-based solutions to address these challenges.

Sequencing of microbial genomes, a task that once took large sequencing centers months to complete, is regularly performed by small laboratories in a few days due to the development of benchtop next-generation sequencing (NGS) platforms. This capability has resulted in a desire to use whole genome sequencing (WGS) for pathogen identification in applied settings such as biothreat detection, molecular epidemiology, and clinical diagnostics. However, a number of challenges related to sample processing and data analysis must be overcome before widespread adoption of WGS is realized.

In an effort to identify priority areas for standards activities and facilitate the development of a measurement infrastructure for NGS-based pathogen identification, the National Institute of Standards and Technology (NIST) convened a two-day workshop composed of representatives from Federal Government, academia, and industry. The workshop took place on October 20–21, 2014 at the NIST campus in Gaithersburg, Maryland. The objectives of the Standards for Pathogen Identification via Next-Generation Sequencing (SPIN) Workshop were to identify current and anticipated future measurement challenges hindering the implementation of NGS in pathogen identification, and to propose avenues to address these challenges including recommendations for standards development (see NIST Special Publication SP1183 for a detailed description of the workshop including summaries of the presentations) [1].

On the first day of the workshop, leaders in the field presented their efforts and thoughts on WGS challenges related to specific areas including metrology, sample preparation, molecular epidemiology, antimicrobial resistance surveillance, large-scale genome sequencing projects, genome sequence databases, bioinformatics methods, and biomarker development (Table 1). These application areas included strain-level isolate identification and discrimination as well as culture-independent diagnostics from complex samples. (The slides for a number of the presentations are available online [2]). Despite the diversity of topics, several recurring themes were identified including:

- the need for standard methods and reference materials for sample processing and data analysis,
- performance metrics for validating data analysis methods,
- well curated and diverse databases, and
- guidance on results interpretation.

**Table 1:** Workshop speakers and presentation titles

Speaker	Presentation Title
David Duewer, NIST	*Metrology for Identity and Other Nominal Properties*
Peter Vallone, NIST	*Forensics: Human Identity Testing in the Applied Genetics Group*
Javier Atencia, University of Maryland	*Separation of Bacteria from Complex Samples*
Nathan Olson, NIST	*Microbial Genomics at NIST*
Adam Phillippy, National Biodefense Analysis and Countermeasures Center	*Sequencing and Informatics for Microbial Forensics*
William Klimke, National Institutes of Health	*Bacterial Pathogen Genomics at NCBI*
Rebecca Lindsey, Centers for Disease Control and Prevention	*Next-Generation Sequencing for Identification and Subtyping of Foodborne Pathogens*
Eric Brown, Food and Drug Administration (FDA)	*Validation, Standardization, and Application of the FDA WGS Pipeline for Foodborne Contamination Traceability*
William Wolfgang, New York State Department of Health	*The Prospects for Nextgen Surveillance of Pathogens: A View from a Public Health Lab*
Patrick McDermott, FDA	*Whole Genome Sequencing and Antibiotic Resistance Surveillance*
Heike Sichtig, FDA & Luke Tallon, University of Maryland	*Development of FDA MicroDB: A Regulatory-Grade Microbial Reference Database*
David Rasko, University of Maryland	*From Genome to Biomarker: The Path Forward*
Bart Weimer, University of California, Davis	*High Throughput Sequencing Pipeline for Diverse Organisms*

The majority of the second day consisted of small and large group discussions to identify current and future measurement challenges associated with implementation of NGS for pathogen identification and potential solutions for these challenges. The entire process, from sample to answer, is challenging and requires a supporting measurement infrastructure to increase confidence in the final results. Each step in the process has measurement challenges, biases, uncertainties, and application-specific components, requiring measurement solutions and standards. Table 2 describes four primary classes of solutions often used to address measurement challenges.

**Table 2:** Classes of metrology-based solutions to support a measurement infrastructure

Solution classes	Description
Materials	Physical materials used to evaluate or validate components such as new or existing laboratory methods, training protocols, and capabilities. These materials can include reference materials for calibration or method validation and quality control materials for routine assessment of run to run performance.
Data	Data used to evaluate and validate bioinformatics pipelines and algorithms.
Guidance documents	Community accepted guidance documents such as standard operating procedures, standard guidance, or standard methods. These documents could be formal voluntary consensus standards or community accepted best practices.
Interlaboratory studies	Results from interlaboratory studies, where a common sample or protocol is distributed to participants for analysis, are used to validate the measurement process and participant’s ability to perform the measurement.

During the workshop, several specific examples of standards-based solutions were discussed. Some of these solutions were identified as potential next steps for standards development based upon their anticipated high impact, expected usage, and relative ease of preparation as compared to other activities. This list is not exhaustive but serves to identify examples related to the four categories of measurement-based solutions.

- *Reference data:* Workshop attendees discussed that development of *in silico* reference data might be a simpler starting point than physical reference materials (such as cells or DNA). Datasets could be created by combining known genome sequences and used to benchmark and compare bioinformatics pipelines and results reporting and interpretation. These datasets could include relevant pathogens as well as environmental contaminants, such as host DNA, other microorganisms, and viral genomes.
- *Reference materials:* Well-characterized microbial genomic DNA reference materials are needed for validation of sequencing platforms, library preparation protocols, sequencing chemistries, and base calling algorithms.
- *Guidance documents:* Reference materials specific for each application area are needed, yet development of such a vast set of reference materials is too large for any one organization. Guidance documents describing methods to develop and characterize control materials would enable industry and others to prepare quality control materials (in house development of the materials) with organisms relevant to their specific application space.
- *Interlaboratory studies:* An interlaboratory study to evaluate the contents of a known mixture of well-characterized microbes is needed to support metagenomic analyses. Results from the study would be used to characterize sources of bias and uncertainty associated with metagenomic sequencing, including DNA extraction, sample processing, and data analysis.

The first steps in establishing a measurement infrastructure for pathogen identification using NGS are already underway. For instance, proposals for documentary standards related to NGS are under consideration by International Organization for Standardization (ISO) TC34: Food Products. These proposals include a standard for foodborne pathogen strain typing using WGS and a more general standard on NGS quality analysis. In a separate effort, NIST in collaboration with the FDA is developing genomic DNA reference materials for four bacterial strains, *Salmonella enterica* subsp. *enterica* serovar Typhimurium strain LT2, *Staphylococcus aureus* clinical isolate, *Pseudomonas aeruginosa* clinical isolate, and *Clostridium sporogenes* PA3679. These materials will be used to validate sequencing chemistries and platforms and to support laboratory proficiency testing. Additionally, data generated from the material can be used to validate bioinformatics workflows such as *de-novo* assembly and variant calling.

Overall, the solutions to the challenges identified during the workshop will serve as the basis for a measurement infrastructure for pathogen identification using WGS. NIST is in a unique position to help advance this field by providing expertise in metrology (measurement science) and by leveraging experience developing reference materials and measurement infrastructures for related fields, such as human genome sequencing [3] and transcriptome sequencing [4]. A similar measurement infrastructure for pathogen identification using NGS will increase confidence in results and improve data informed decision-making, in turn enabling WGS to achieve its full potential in revolutionizing pathogen identification.

## Abbreviations

NGS: Next Generation Sequencing
WGS: Whole Genome Sequencing
NIST: National Institute of Standards and Technology
ISO: International Organization for Standards
DNA: Deoxyribonucleic Acids
FDA: Food and Drug Administration
NCBI: National Center for Biotechnology Information
